# Monocyte interaction accelerates HCl-induced lung epithelial remodeling

**DOI:** 10.1186/1471-2466-14-135

**Published:** 2014-08-09

**Authors:** Qiuhua Chen, Alice Aili Luo, Haibo Qiu, Bing Han, Bruce Hsin-Kuo Ko, Arthur S Slutsky, Haibo Zhang

**Affiliations:** 1 Keenan Research Center for Biomedical Science of St. Michael’s Hospital, Toronto, Ontario, Canada; 2 Department of Critical Care Medicine, Nanjing Zhong-Da Hospital, Southeast University School of Medicine, Nanjing, PR China; 3 Department of Respiratory Therapy, Taipei Veterans General Hospital and Institute of Physiology, School of Medicine, National Yang-Ming University, Taipei, Taiwan; 4 Interdepartmental Division of Critical Care Medicine, University of Toronto, Toronto, Ontario, Canada; 5 Department of Anesthesia and Department of Physiology, University of Toronto, Room 619, LKSKI, 209 Victoria St, Toronto, Ontario M5B 1T8, Canada

**Keywords:** ARDS, PDGF, Chemokine, EMT

## Abstract

**Background:**

Acute respiratory distress syndrome (ARDS) is characterized by overwhelming inflammatory responses and lung remodeling. We hypothesized that leukocyte infiltration during the inflammatory response modulates epithelial remodeling through a mechanism of epithelial-mesenchymal transition (EMT).

**Methods:**

Human lung epithelial cells were treated for 30 min with hydrochloric acid (HCl). Human monocytes were then cocultured with the epithelial cells for up to 48 h, in the presence or absence of blocking peptides against lymphocyte function-associated antigen-1 (LFA-1), or tyrphostin A9, a specific inhibitor for platelet-derived growth factor (PDGF) receptor tyrosine kinase.

**Results:**

Exposure of lung epithelial cells to HCl resulted in increased expression of intercellular adhesion molecule-1 (ICAM-1) and production of interleukin (IL)-8 at 24 h. The expression of the epithelial markers E-cadherin decreased while the mesenchymal markers vimentin and α-smooth muscle actin (α-SMA) increased at 24 h and remained high at 48 h. The addition of monocytes augmented the profiles of lower expression of epithelial markers and higher mesenchymal markers accompanied by increased collagen deposition. This EMT profile was associated with an enhanced production of IL-8 and PDGF. Treatment of the lung epithelial cells with the LAF-1 blocking peptides CD11a_237–246_ or/and CD18_112–122_ suppressed monocyte adhesion, production of IL-8, PDGF and hydroxyproline as well as EMT markers. Treatment with tyrphostin A9 prevented the EMT profile shift induced by HCl stimulation.

**Conclusions:**

The interaction between epithelial cells and monocytes enhanced epithelial remodelling after initial injury through EMT signalling that is associated with the release of soluble mediators, including IL-8 and PDGF.

## Background

Acute respiratory distress syndrome (ARDS) continues to have a very high mortality in critically ill patients [1,2]. Lung remodeling may take place early in the course of ARDS and is associated with decreased quality of life and poor outcome [3,4]. Pulmonary fibrosis was found in open-lung biopsies of 53% of ventilated patients who had ARDS for ≥ 5 days, and their mortality rate was 57% as compared to 0% in ARDS patients who did not have fibrosis [1,4,5]. A more recent study has confirmed that the severity of pulmonary fibrosis can predict mortality and ventilator dependency in patients with ARDS [6]. We have recently demonstrated in a mouse model of acid aspiration (HCl)-induced ARDS that epithelial-mesenchymal transition (EMT) is an important signaling pathway contributing to the development of pulmonary fibrosis [7].

Alveolar macrophages play a major role in the pathogenesis of acid aspiration-induced lung injury [8]. In animal models of ARDS, crosstalk between macrophages and structural cells contributes to ongoing inflammatory responses [9]. Moreover, the activation of macrophages may promote pulmonary fibrosis as evidenced by a recent study [10] showing that a greater number of fibroblasts was associated with a larger percentage of macrophages in lung lavage of ventilated patients with ARDS. Similarly, coculture of monocytes with human tubular epithelial cells resulted in the development of fibrotic like phenotypes in association with upregulation of intercellular adhesion molecule-1 (ICAM-1) [11]. Taken together, these data suggest that alveolar macrophages may not simply be innocent bystanders but may play an important role in pulmonary remodeling.

To examine the hypothesis that macrophages modulate lung epithelial cell remodeling through a mechanism of EMT, we developed an *in vitro* model of human lung epithelial cell injury induced by administration of HCl followed by coculture with human monocytes to mimic leukocyte infiltration. We demonstrate that the interaction of macrophages/monocytes with the injured epithelial cells accelerates lung epithelial remodeling characterized by EMT profiles through direct cell-cell contact and the release of platelet-derived growth factor (PDGF).

## Methods

### Cells

Primary human small airway epithelial cells (SAEC, Lonza Group, Basel, Switzerland) were grown as monolayer in 5% CO2 at 37°C in SAGM (Lonza Group, Basel, Switzerland) with 50 μg/ml penicillin, 50 mg/ml streptomycin (Sigma, St. Louis, MO) and 10% heat-inactivated FBS (Sigma, St. Louis, MO). Human lung epithelial cells (BEAS-2B, ATCC, Manassas, VA) were grown as monolayer in 5% CO_2_ at 37°C in DMEM with 50 μg/ml penicillin, 50 mg/ml streptomycin (Sigma) and 10% heat-inactivated FBS (Sigma). Primary human peripheral monocytes (Stem Cell Technologies, Vancouver, BC, Canada) or human monocyte line (U937, ATCC) were grown in suspension in 5% CO_2_ at 37°C in RPMI-1640 with 50 μg/ml penicillin, and 50 mg/ml streptomycin (Sigma) and 10% heat-inactivated FBS (Sigma).

### Exposure of lung epithelial cells to HCl

SAEC were seeded in 12-well plate (BD Biosciences, Bedford, MA) at a density of 2.5 × 10^5^ cells/well in HCl group or 1.75 × 10^5^ cells/well in vehicle control group for 24 h. BEAS-2B cells (4 × 10^5^ or 1 × 10^5^ cells/well) were placed in 6-well or 24-well plates (BD Biosciences, Bedford, MA), respectively. After 24 h of incubation, the medium was replaced with serum-free SAGM or DMEM for 24 h. The cells were then treated with serum-free SAGM or DMEM either alone (Vehicle, pH 7.4) as control, or containing 40 mM HCl (HCl, pH 4.0) at 37°C in 5% CO_2_, for 30 min. The acidified medium was discarded and the cells were washed three times with complete SAGM or DMEM medium. Then the cells were cultured for up to 48 h.

### Blocking peptides

In separate experiments, SAEC or BEAS-2B cells were incubated with the blocking peptides CD11a_237–246_ (sequence: ITDGEATDSG derived from the residues I237-246 of the 190 amino acid I-domain of the LFA-1 α-subunit), CD18_112–122_ (sequence: DLSYSLDDLR derived from residues D134-Q159 of the LFA-1 β-subunit) [12] at a dose of 0.1 or 1 mM, or tyrphostin A9, a specific inhibitor for PDGF receptor tyrosine kinase (Cayman Chemical, Ann Arbor, MI) at doses of 5 and 10 μM, 30 min prior to coculture with primary human monocytes or U937 cells (6.25 × 10^4^ cells/well for 30 min). The scrambled peptide (sequence: ITDDAGTGSE) served as a control (GenScript USA Inc. Piscataway, NJ).

### Cell death assay

Cell viability was evaluated before and after HCl administration by using a cytotoxicity assay (Roche GmbH, Mannheim, Germany). Briefly, lactate dehydrogenase (LDH) was measured at 490 nm (SpectraMax M5, Molecular Devices). In pilot studies, the LDH level increased by 30% after incubation for 30 min with HCl at the dose used in the epithelial cells. In the subsequent experiments, a cell density of 30% higher than the vehicle control was seeded in the HCl group in order to reach a similar cell density as seen in the saline control group before the coculture with monocytes.

### Monocyte adhesion assay

Confluent BEAS-2B cells were incubated in serum-free DMEM for 24 h, and treated with either or DMEM containing HCl at pH 4.0 or DMEM at pH 7.4 for 30 min. After medium replacement and 3 washes with DMEM, U937 cells (10^5^ cells) that were loaded for 30 min with 5 μM calcein-AM (Molecular Probes, Inc., Eugene, OR) were added onto the monolayer of the BEAS-2B cells and cocultured for 48 h. Monocyte adhesion was evaluated by measuring calcein-AM density from six randomly selected, high-power fields using a fluorescent microscope (Nikon Eclipse E800) [13]. This was performed by two independent investigators, blinded to the study groups.

### Western blotting

Total proteins were obtained from SAEC or BEAS-2B cells after different treatments, separated by 10% SDS-PAGE gel under reducing conditions, and then transferred to polyvinylidenedifluoride membranes. The membranes were blocked with 5% bovine serum albumin in TBS (20 mM Tris, pH 7.5, and 150 mM NaCl) containing 0.1% Tween-20 for 1 h, then probed with antibodies against human ICAM-1, E-cadherin, vimentin, cytokeratin 8, N-cadherin (Santa Cruz Biotechnology, Santa Cruz, CA), and α-smooth muscle actin (α-SMA) (Abcam, Cambridge, UK), respectively in primary antibody dilution buffer over night at 4°C. After washing, the membranes were incubated with appropriate secondary antibodies conjugated with horse-radish peroxidase (Jackson ImmunoResearch Lab, West Grove, PA), and the signals were detected with an enhanced chemiluminescence kit (Pierce, Rockford, IL).

### IL-8 and platelet-derived growth factor (PDGF) assays

Concentrations of IL-8 and PDGF in the supernatant were measured using enzyme-linked immunosorbent assay kits (BD Bioscience, Bedford, MA and Abcam Inc. Cambridge, MA, respectively).

### Hydroxyproline assay

The cell culture supernatants were dried at 100°C for 48 h followed by addition of NaOH (150 μl, 4 M), kept in boiling water for 90 min. In order to change the pH of hydrolysate to 6.0, citric acid (150 μl, 1.4 M) was added, followed by addition of Chloramine-T solution and incubated at room air for 20 min. Then Erlich’s solution at 1 ml [2.5 g 4-(dimethylamino) benzaldehyde, 9.3 ml n-propanol, and 3.9 ml 70% perchloric acid] was added and incubated for 20 min at 65°C. A standard curve was constructed [14]. Absorbance was measured at 560 nm.

### Statistical analysis

Data are expressed as mean ± SEM. One-way ANOVA followed by the Tukey/Kramer test was used for statistical analysis. Differences were considered statistically significant at *P* values < 0.05.

## Results

### Acid exposure model of lung epithelial cell injury and EMT

**Figure 1:**
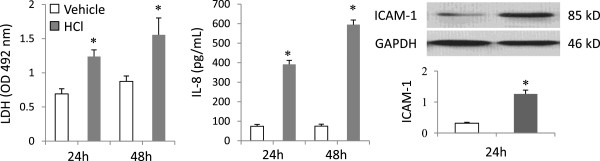
**HCl induced lung epithelial injury and inflammatory responses.** Human BEAS-2B cells were treated for 30 min with serum-free DMEM either alone (Vehicle, pH 7.4) or containing 40 mM HCl (HCl, pH 4.0). The acidified medium was discarded and the cells were washed three times with complete DMEM to confirm neutralization of the culture medium. The cells were then cultured for up to 48 h. LDH and IL-8 were measured in culture supernatants. ICAM-1 expression was evaluated by Western Blot in cell lysates. Data shown are in triplicate from three experiments. ^*^P < 0.05 vs. Vehicle at identical time point, respectively.

**Figure 2:**
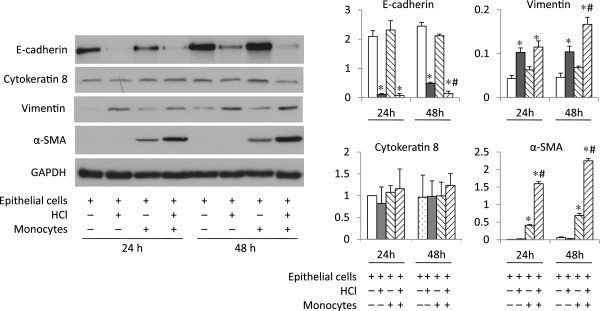
**Interactions between monocytes and lung epithelial cells resulted in increased EMT profiles following HCl challenge.** Confluent human BEAS-2B cells were treated for 30 min with either DMEM at pH 4.0 containing HCl or DMEM at pH 7.4. After medium replacement and wash, U937 cells (10^5^ cells) were added onto the monolayer of the BEAS-2B cells and cocultured for up to 48 h. Expression of E-cadherin, vimentin and α-SMA was detected by Western blots. Representative blots are shown in left panel. Semi-quantative data of the band density from three experiments are shown in right panel. ^*^P < 0.05 vs. vehicle control (open bar) and ^#^P < 0.05 vs. Epithelial cells + Monocytes at identical time point, respectively.

The HCl challenge for 30 min resulted in a significant increase in LDH release from the epithelial cells at 24 h (Figure 1); cell viability was 81.6 ± 1.2% evaluated by Trypan Blue Exclusion at 24 h. LDH levels increased further at 48 h but the difference did not reach statistically significance compare to 24 h, and remained stable thereafter at 72 h (Data not shown in pilot studies). HCl stimulation increased IL-8 production (Figure 1) as well as expression of the co-stimulatory molecule ICAM-1 (Figure 1) in the lung epithelial cells.Stimulation of the lung epithelial cells with HCl resulted in decreased expression of E-cadherin and increased expression of vimentin; α-SMA was unaffected at 24 h. The EMT pattern remained constant for 48 h after HCl challenge (Figure 2).

### Monocyte adhesion enhanced EMT profiles

**Figure 3:**
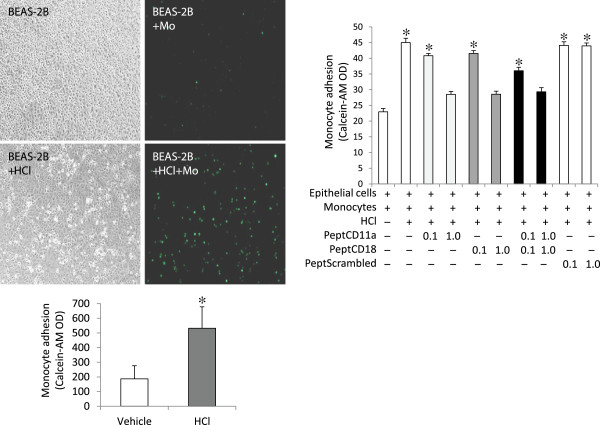
**HCl treatment enhanced monocyte adhesion to lung epithelial cells via crosstalking between LAF-1 and ICAM-1.** Human BEAS-2B cells were treated for 30 min with serum-free DMEM either alone (pH 7.4) or containing HCl (pH 4.0). The medium was replaced with complete DMEM at pH 7.4. The cells were then incubated with the LFA-1 peptide CD11a_237–246_ (PeptCD11a), CD18_112–122_ (PeptCD18), or both in combination (PeptCD11a + CD18) with scrambled peptides (PeptScrambled) as control at 0.1 or 1.0 mM for 30 min, human U937 cells (10^5^ cells) labeled with Calcein-AM were then added onto the monolayer of the BEAS-2B cells and cocultured for 48 h. Monocyte adhesion was evaluated by measurement of Calcein-AM density. Data shown are in triplicate from three experiments. ^*^P < 0.05 vs. vehicle control (far left bar).

**Figure 4:**
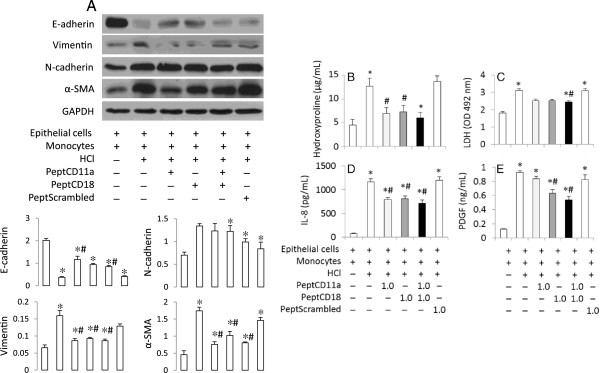
**Blockade of monocyte-epithelial cell interaction attenuated EMT profiles and inflammatory responses.** Human BEAS-2B cells were treated for 30 min with serum-free DMEM either alone (pH 7.4) or containing HCl (pH 4.0). The medium was replaced with complete DMEM at pH 7.4. The cells were then incubated for 30 min with the LFA-1 peptide CD11a_237–246_ (PeptCD11a), CD18_112–122_ (PeptCD18), combination of CD11a_237–246_ and CD18_112–122_ (PeptCD11a + CD18) or scrambled peptides (PeptScramb) at 1.0 mM. U937 cells were added onto the monolayer of the BEAS-2B cells and cocultured for 48 h. **A**. Expression of epithelial markers of E-cadherin, and mesenchymal markers of vimentin, α-SMA and N-cadherin were detected by Western blots. Upper panel shows representative blots. Lower panel shows semi-quantative data of the band density from three experiments. ^*^P < 0.05 vs. vehicle control (Control); ^#^P < 0.05 vs. HCl alone, respectively. **B**-**E**. Cell viability and inflammatory responses by monocyte-epithelial cell interaction following HCl challenge. Concentrations of hydroxyproline, LDH, IL-8 and PDGF were measured in culture supernatants. Data shown are in triplicate from three experiments. ^*^P < 0.05 vs. vehicle control (far left bar); ^#^P < 0.05 vs. HCl alone, respectively.

We then examined the impact of the increased ICAM-1 expression by lung epithelial cells following exposure to HCl by focusing on monocyte adhesion. As shown in Figure 3, monocyte adhesion to the epithelial cells that were previously exposed to HCl increased dramatically, compared to epithelial cells exposed to control buffer.The enhanced monocyte adhesion was most likely explained by increased expression of ICAM-1 in epithelial cells after HCl stimulation (Figure 1), given that administration of CD11a/CD18 blocking peptides, but not the scrambled peptide, attenuated cell adhesion in a dose-dependent manner (Figure 3).The enhanced monocyte adhesion to epithelial cells resulted in greater changes in the markers of EMT profiles compared to lung epithelial cells alone. The expression of E-cadherin decreased while vimentin and α-SMA increased dramatically at 48 h, compared to either the epithelial cell alone after HCl stimulation (Figure 2), or the co-culture of epithelial cells with monocytes without HCl challenge (Figure 4A). The phenotypic changes of EMT profiles were associated with increased collagen deposition as reflected by a higher hydroxyproline concentration 48 h after HCl challenge (Figure 4B).

### IL-8 and PDGF responses

We examined the mechanisms by which monocytes contributed to EMT profiles in HCl-stimulated lung epithelial cells by evaluation of cell death, IL-8 production and expression of PDGF in the coculture conditions. Epithelial damage is an important initial event in pulmonary fibrosis; in our model greater cell death was reflected by higher LDH release after HCl stimulation (Figure 4C). Since IL-8 is involved in monocyte adhesion and lung remodeling [15,16], we measured IL-8 production and found that it increased in the culture supernatants (Figure 4D). A mechanism by which activated monocytes accelerate lung fibrosis has been reported through upregulation of PDGF [17]. The latter also induces IL-8 production in primary human lung cells [18]. We noted a dramatic increase in the concentration of PDGF after HCl stimulation in the coculture system (Figure 4E).

### Inhibition of CD11a and CD18 attenuated EMT

**Figure 5:**
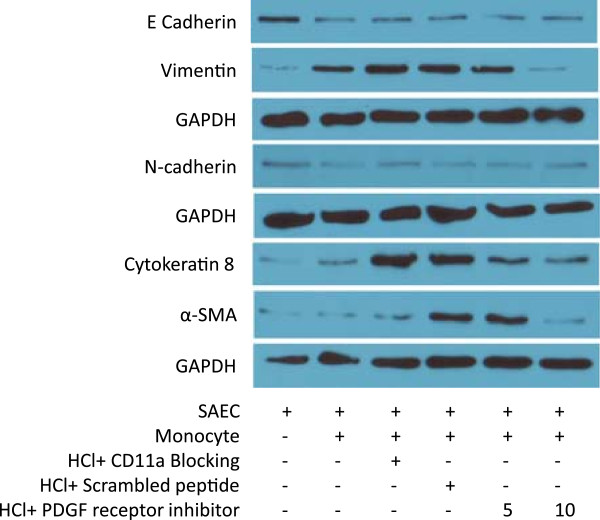
**Blockade of monocyte-epithelial cell interaction or PDGF receptor attenuated EMT profiles.** Primary human SAEC were treated for 30 min with serum-free SAGM either alone (pH 7.4) or containing HCl (pH 4.0). The medium was replaced with complete SAGM at pH 7.4. The cells were then incubated for 30 min with tyrphostin A9, a specific inhibitor for PDGF receptor tyrosine kinase at 5 or 10 μM. Primary human monocytes were added onto the monolayer of the SEAC and cocultured for 48 h. Expression of EMT markers was detected by Western blots.

It appears that both the epithelial-monocyte contact and the released soluble mediators played a role in accelerating the EMT phenotypes after HCl challenge. We thus examined the possibility of attenuating the EMT changes by blocking the cell contact-initiated inflammatory event. The presence of the single blocking peptide with either CD11a_237–246_ or CD18_112–122_ attenuated cell adhesion in a dose-dependent manner while the combination of the two peptides did not induce any further effect (Figure 3). The high dose of the peptides was used in the subsequent studies reported in Figures 4 and 5.

The treatment of epithelial cells with CD11a_237–246_ or CD18 CD18_112–122_ peptides maintained a higher level of expression of E-adherin at the protein level, and reduced expression of vimentin and α-SMA at 48 h in the coculture conditions after HCl stimulation (Figure 4A). Combining both peptides did not enhance the effects compared to application of either of the individual peptides (Figure 4A). The changes of EMT profiles were concomitant with changes of the level of cell death as reflected by LDH concentration, collagen deposition of hydroxyproline, and production of IL-8 and PDGF (Figure 4B-E).Similar to those observed using the blocking peptides, the treatment of primary human SAEC with the inhibitor specific for PDGF receptor tyrosine kinase, attenuated EMT profile shift after HCl challenge in the coculture conditions with primary human monocytes (Figure 5).

## Discussion

The main findings of this study are: 1) we established an *in vitro* model of acid-induced lung epithelial cell injury, inflammatory responses, and epithelial remodeling; 2) we demonstrated that interaction of monocytes with preexisting injured lung epithelial cells resulted in accelerated epithelial remodeling toward EMT phenotypes; and 3) the mechanisms by which monocytes promoted EMT profiles appeared to be direct cell-cell contact with subsequent release of soluble mediators including IL-8 and PDGF.

Gastric aspiration frequently takes place in trauma or ICU patients when the status of consciousness is altered [19]. In a prospective study of critically ill, it has been shown that at least one aspiration event occurred in 89% of patients using lung lavage fluid levels of pepsin as an indicator of aspiration [20]. In particular, acid aspiration pneumonia is a well-known risk factor for ARDS [21]; the injury is characterized by leukocyte infiltration, which can lead to pulmonary fibrosis [22].

Instillation of HCl directly into the trachea or bronchi is a well-established animal model [23]. A feature of this lung injury model is plasma membrane disruption of the alveolar epithelium, including type II alveolar epithelial cells [24,25]. Acid aspiration in animal models caused rapid migration of monocytes into the lungs after injury [19], followed by fibrotic formation and reduced lung compliance, suggesting that the alveolar epithelium is a key target site of acid aspiration [23]. However, given the multitude of cell types in the lung [26], it is difficult *in vivo* to specifically examine mechanisms of lung remodeling associated with epithelial cells, especially the EMT pathways.

To establish an *in vitro* model of lung epithelial cell remodeling, we exposed human lung epithelial cells to HCl for 30 minutes, followed by observation of the epithelial repair process for 48 hours. The HCl-induced epithelial cell injury and inflammatory responses were characterized by altered cell viability, increased expression of ICAM-1 and production of IL-8. Epithelial cell injury is followed by remodeling processes, which consist of epithelial and mesenchymal activation, cytokine production, activation of growth factor pathways, re-epithelialization and fibrosis [27]. We observed that the cell repair processes after HCl insult were associated with epithelial remodeling characterized by the changes of EMT phenotypes. This finding is consistent with our previous *in vivo* study demonstrating that pulmonary EMT took place after intratracheal instillation of HCl in mice [7].

Another recent study demonstrated massive recruitment of neutrophils and macrophages into the lung that was associated with fibrotic evolution in a murine, HCl-induced lung injury model but the mechanisms involved were not investigated [28]. The present *in vitro* model mimics the *in vivo* conditions, and provides mechanistic insights demonstrating that the interaction of monocytes with the injured lung epithelial cells after HCl challenge accelerates the lung remodeling process.

There are several mechanisms to explain the increased lung epithelial remodeling after interaction with monocytes: 1) Direct cell-cell contact mediated by ICAM-1 was required to initiate activation of monocytes by injured lung epithelial cells. In lung, activated macrophages are generally characterized as M1 and M2 phenotypes based on the patterns of their inflammatory responses. The M2 alveolar macrophages activate STAT3 signaling leading to fibrotic formation [29]. The role of macrophages in promoting pulmonary fibrosis was reported in a recent study [10] showing that recovery of higher numbers of fibroblasts was associated with a higher percentage of macrophages in lung lavage of ventilated ARDS patients. However, the mechanisms by which macrophages participated in pulmonary fibrotic formation were not addressed [10]; 2) The enhanced production of IL-8 by the activated monocytes plays an important role. IL-8 is a C-X-C chemokine that contributes to acute inflammation through its G protein-coupled receptors CXCR1 and CXCR2. It has been reported that IL-8 acts as an autocrine regulator of IL-8 production and its signaling in human monocytes through mitogen-activated protein kinase (MAPK) pathways [30]. Increased expression of IL-8 by alveolar macrophages likely contributes to the activity of pulmonary fibrotic formation [31]. IL-8 has also been shown to promote EMT in human carcinoma cells [32]; and 3) Increased release of soluble PDGF as a result of the interaction between monocytes and the HCl-challenged lung epithelial cells. It is known that IL-8 production is mediated by PDGF in many cell types [18,33,34]. In addition, PDGF has been long considered to be a profibrotic cytokine [35]. The mechanisms were unknown until recent studies suggesting that PDGF mediates EMT in epicardial [36] and cancer cells [37].

In the present study, we used the short peptides derived from LFA-1, a family of leukocyte integrins including the common β-chains (β2, CD18) and a distinct α-chain (αL, CD11a) that binds to ICAM-1. The peptides have been used to interfere with the binding activity and thus inhibition of epithelial-leukocyte adhesion [38]. It has been shown that these N-terminal fragments of CD11a_237–246_ derived from the residues I237-246 of the 190 amino acid I-domain of the LFA-1 α-subunit) and CD18_112–122_ from residues D134-Q159 of the LFA-1 β-subunit) more efficiently inhibited the binding to ICAM-1 and mixed lymphocyte reaction than the parent peptides, and they are as effective as, or more effective than the blocking anti-LFA-1 antibodies at concentrations of 600 μM and above [12]. Our results suggest that inhibition of the interaction between monocytes and the HCl-challenged lung epithelial cells can attenuate the epithelial remodeling toward EMT. Since this attenuation was associated with decreased production of both IL-8 and PDGF in the presence of the blocking peptides, we believe that cell-cell contact might have played an essential role in the inflammatory responses that are responsible for the accelerated EMT after epithelial injury.

We further examined the effects of a cell-permeable, potent, selective, and ATP-competitive inhibitor of the PDGF receptor family of tyrosine kinases in the coculture conditions of primary human lung epithelial cells and monocytes following HCl challenge. An attenuation of EMT profiles was revealed by using the inhibitor, suggesting the role of PDGF in contributing to EMT. Our results are in agreement with the observation in breast cancer cells where PDGF has been reported to mediate EMT [37].

The neutralization of soluble mediators such as IL-8 and PDGF could be another option to examine the individual role of the mediators on EMT. However, it would not block ongoing release of mediators as opposed to stopping cell-cell contact. The study was conducted *in vitro*, suggesting a very interesting potential mechanism in epithelial remodeling which may have implications for ARDS. Further *in vivo* studies are required to confirm the findings.

## Conclusion

In conclusion, our results demonstrate that acid stimulation induces lung epithelial cell remodeling toward EMT phenotypes, and that leukocytes can promote this remodeling process by release of soluble mediators. The effects of leukocytes on lung epithelial remodeling can be largely inhibited by using LFA-1 blocking peptides or PDGF receptor tyrosin kinase inhibitor. These results suggest possible targets to block the pulmonary fibrotic process by inhibition of epithelial cell and macrophage interaction in inflammatory conditions.

## Competing interests

The authors declare that they have no competing interests.

## Authors’ contributions

QC carried out the cell and molecular studies and drafted the manuscript and performed the statistical analysis. AAL conducted the primary human cell experiments. BH and BHKK participated in the cell culture study and carried out the immunoassays. BH and HQ participated in the revision of the manuscript. AS and HZ participated in the design of the study and revision of the manuscript. All authors read and approved the final manuscript.

## Pre-publication history

The pre-publication history for this paper can be accessed here:

http://www.biomedcentral.com/1471-2466/14/135/prepub
